# Syntaxins on granules promote docking of granules via interactions with munc18

**DOI:** 10.1038/s41598-017-18597-z

**Published:** 2018-01-09

**Authors:** Maria Borisovska

**Affiliations:** Department of Physiology & Pharmacology, Oregon Health & Science University, Portland, Oregon United States

## Abstract

SNAREs and SNARE-binding accessory proteins are believed to be central molecular components of neurotransmitter release, although the precise sequence of molecular events corresponding to distinct physiological states is unclear. The mechanism of docking of vesicles to the plasma membrane remains elusive, as the anchoring protein residing on vesicles is unknown. Here I show that targeting small amounts of syntaxin to granules by transmembrane domain alteration leads to a substantial enhancement of syntaxin clustering beneath granules, as well as of morphological granule docking. The effect was abolished without munc18 and strongly reduced by removal of the N-terminal peptide in the syntaxin mutant. Thus, in contrast to the current paradigm, I demonstrate that syntaxin acts from the vesicular membrane, strongly facilitating docking of vesicles, likely via interaction of its N-peptide with munc18. Docking was assayed by quantifying the syntaxin clusters beneath granules, using two-color Total Internal Reflectance Fluorescence microscopy in live PC-12 cells and confirmed by electron microscopy. Hereby, I propose a new model of vesicle docking, wherein munc18 bridges the few syntaxin molecules residing on granules to the syntaxin cluster on the plasma membrane, suggesting that the number of syntaxins on vesicles determines docking and conceivably fusion probability.

## Introduction

Brain functions are carried out by means of chemical neurotransmission. Vesicles filled with neurotransmitter molecules fuse with the plasma membrane at the presynaptic side, releasing their contents into the synaptic cleft. Then, neurotransmitter molecules diffuse and bind to the receptors on the postsynaptic membrane of a different neuron, thus transmitting the signal. Neurotransmission is very quick: to meet the speed requirement of hundreds of microseconds, several distinct preparatory steps occur prior to final neurotransmitter release. First, vesicles attach to the plasma membrane in a process called docking; subsequently, a calcium-dependent priming process transitions vesicles into the release-ready state, such that prior to fusion vesicles are arrested in a final state^1^. The exact sequence of molecular events underlying each step of neurotransmitter release is still unclear.

SNARE proteins are critical for neurotransmitter release, as demonstrated by neurotoxin cleavage or genetic deletion of SNAREs, which leads to almost complete loss of neurotransmission^2,3^. The SNARE protein family consists of 3 proteins, with syntaxin and SNAP-25 predominantly found on the plasma membrane, while most of synaptobrevin is found on vesicles. It is believed that synaptobrevin binds to SNARE protein counterparts on the plasma membrane: syntaxin and SNAP-25, attaching a vesicle to the plasma membrane and ultimately driving membrane fusion. The defining feature of SNAREs is an ability to form a very tight complex that has the energetic capacity to overcome membrane repulsive forces and drive membrane fusion^4^.

Docking of vesicles was first observed in electron microscopy and interpreted as attachment of vesicles to the plasma membrane. Docking of vesicles is prerequisite for exocytosis. However, the molecular mechanism of docking remains a mystery. The cytoplasmic SNARE accessory protein munc18, which binds to syntaxin with high affinity, appears to be critical for docking^5^. Genetic deletion or changes in expression levels of munc18 have direct effects on the docking of secretory granules and synaptic vesicles^6–9^. Syntaxin is believed to be the plasma membrane’s docking acceptor molecule; prior to fusion, vesicles recruit syntaxin. In neuroendocrine cells, syntaxin molecules form clusters on the plasma membrane where granules dock and fuse^10–13^. Similar syntaxin clustering at release sites was also shown in Drosophila neuromuscular junction^14,15^. Association of a granule with a syntaxin cluster as measured by Total Internal Reflection Fluorescence (TIRF) microscopy on the plasma membrane was referred to as *molecular docking* by Barg *et al*., 2010. Gandasi and Barg 2014 show that docking coincides with formation of syntaxin1/munc18 clusters at the nascent docking site, providing additional evidence that clustering of syntaxin can be used as a measure for vesicle docking. However, in TIRF microscopy, the distance between granules and the plasma membrane is not measured. Therefore the term *molecular docking* should not be confused with the morphological term *docking*, which is derived from electron microscopy and is typically measured as distance between the vesicles and the plasma membrane.

The protein that ties a granule to the acceptor docking complex on the plasma membrane remains elusive. Synaptobrevin and synaptotagmin, which are primarily found on vesicles and granules, were proposed to bear such a role. However, removal or cleavage of those proteins neither affected morphological docking assayed by electron microscopy nor molecular docking assayed by syntaxin cluster formation beneath granules^16,17,12^.

The crystal structure of syntaxin-munc18 complex revealed multiple syntaxin domains binding to munc18. Because those two binding domains are spatially segregated it seemed possible that a single munc18 molecule can bind to two syntaxins^18,19^. Moreover, low quantities of syntaxin are also found on synaptic vesicles and granules^20–22^. Thus, it led me to hypothesize that the docking counterpart on granules is syntaxin itself. Investigation of syntaxin function is difficult; deletion of this protein and its isoforms is prohibitive, due to its critical role in development and to the viability of cells^23–25^. To test my hypothesis, I increased the number of syntaxin molecules on granules by altering the transmembrane domain of syntaxin with a corresponding domain of synaptobrevin 2. TIRF allows visualization of fluorescent molecules at a very shallow depth from cell surface. By quantifying formation of syntaxin clusters beneath granules in live PC-12 cells I quantified *molecular docking* of secretory granules to the plasma membrane. Electron microscopy was employed to validate that the observed enhancement of syntaxin recruitment corresponds to enhancement of *morphological granule docking* to the plasma membrane.

## Results

The PC-12 cell line derived from rat adrenal medulla was used for experiments. PC-12 cells exhibit regulated exocytosis and express all the major synaptic proteins required for regulated neurotransmitter release. Simultaneous dual-color TIRF microscopy was used to visualize granules in close proximity to the plasma membrane (<150 nm) and quantify syntaxin clusters beneath granules, similar to as previously described^12^. In my experiments, Syntaxin1A was labeled with monomeric GFP linked via a flexible (4xGGS) linker to the C-terminus (Syx-GFP), such that the GFP was exterior to the cell. Syntaxin-GFP was shown to have the same clustering characteristics as the endogenous syntaxin^11,26^. Low expression levels of Syx-GFP were ensured by placing its coding sequence into the second reading frame of bicistronic vector (IRES Syx-GFP). Only cells with Syx-GFP fluorescence intensity ranging from 400–2000 in the footprint were taken, corresponding to an estimated 0.22–1.09 fold expression levels above endogenous syntaxin levels^13^ (Supplementary Fig. 1d). Within this range of expression levels, syntaxin clustering does not depend on the expression levels^13^.

**Figure 1:**
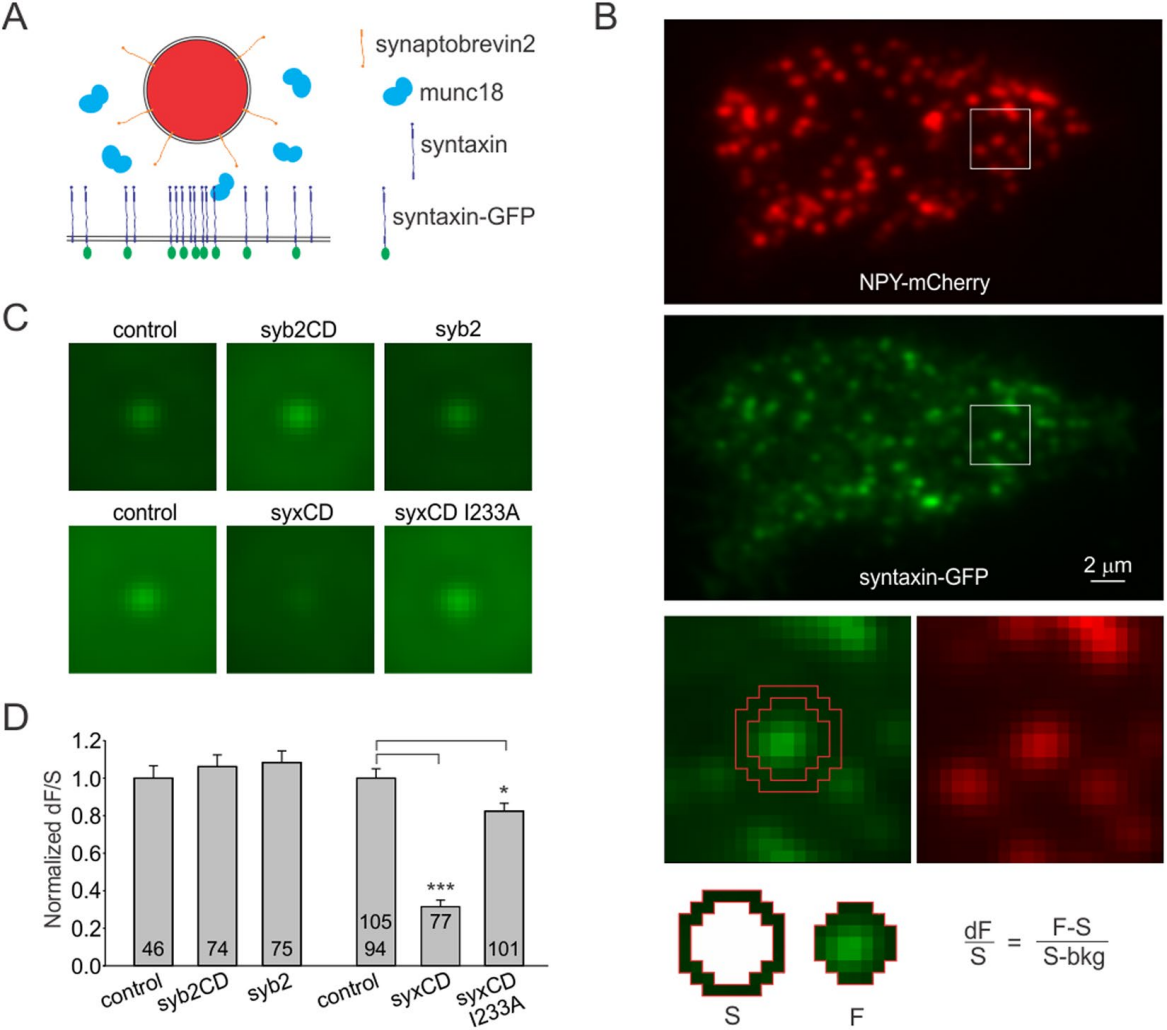
Synaptobrevin 2 has no effect on syntaxin cluster formation. (**A**) Schematic molecular model representing a granule (red) above the plasma membrane with endogenous syntaxin, as well as syntaxin-GFP accumulating beneath granules. (**B**) Exemplary dual-color TIRF image of a live PC-12 cell (#1421-10) expressing NPY-mCherry labeled granules (top panel, scaling: 1800–3500) and syntaxin-GFP (middle panel, scaling: 1800–7000). Bottom panels illustrate analysis of a solitary granule from a boxed area (21 × 21 pxl). GFP fluorescence beneath granule (F) and surrounding annulus (S) are used to quantify syntaxin cluster by calculating dF/S (bkg = background fluorescence). (**C**) Averaged Syx-GFP fluorescence beneath granules (scaled 2300–3700) obtained by averaging 21 × 21 pxl green fluorescent images for all qualifying solitary granules per condition. Overexpression of synaptobrevin 2 (syb2) or cytoplasmic domain of synaptobrevin 2 (syb2CD) did not change Syx-GFP clustering (top panels). Expression of cytoplasmic domain of syntaxin (syxCD) dramatically diminished Syx-GFP cluster formation; I233A mutation strongly diminished that phenotype (syxCD I233A). (**D**) Quantification of cluster formation (dF/S) for conditions shown in **c**, normalized to control values. Number of cells included in analysis is indicated inside the bars. 105 cells served as controls for syxCD, and 94 for syxCD I233A.

As laser light illuminates from underneath the cell surface attached to the coverslip, granules are always above the plasma membrane and syntaxin-GFP signal is beneath granules. Syx-GFP competed with endogenous syntaxin and served as the read-out for syntaxin cluster formation beneath granules (Fig. 1A, schematic drawing). Granules were labeled by Neuropeptide Y linked to mCherry (NPY-mCherry) using a separate plasmid; NPY was shown to be a reliable marker of neurosecretory granules^27^.

Exemplary simultaneous dual red and green image of a cell expressing NPY-mCherry and Syx-GFP is shown in Fig. 1B. The dF/S value was used to quantify syntaxin cluster formation beneath granules, serving as a measure of molecular docking. dF/S was calculated via a simple formula determining brightness of green fluorescence beneath granule (F) compared to fluorescence in the surrounding annulus (S) and normalized to the syntaxin-GFP expression level (Fig. 1B). Syntaxin cluster beneath granules as measured by dF/S does not depend on the syntaxin-GFP expression levels within a broad range of syntaxin-GFP levels^13^. Only solitary granules were included in analysis, thus, the surrounding annulus was a granule-free zone.

### Molecular docking is insensitive to manipulation of synaptobrevin 2

First, I tested whether synaptobrevin 2 has any effect on syntaxin clustering beneath granules. If synaptobrevin is a part of the docking complex, its enrichment would likely lead to enhancement of docking; on the other hand, overexpression of a cytoplasmic domain of synaptobrevin 2 would saturate docking acceptor binding sites, diminishing docking. The TIRF experimental setup only allowed visualization of two colors, and therefore, I expressed unlabeled synaptobrevin 2 (syb2) using a bicistronic vector containing Syx-GFP in the second reading frame after the IRES site (syb2 IRES Syx-GFP). The expression levels of the protein in the first reading frame were estimated to be over 2.8 fold higher than the protein in the second reading frame (Supplementary Fig. 1). The expression of unlabeled proteins was validated using immunocytochistry and western blotting (Supplementary Figures 2 and 3).

As indicated by both the averaged Syx-GFP cluster beneath granules and dF/S analysis; neither the cytoplasmic domain of syb2 (syb2CD, 1–96 amino acids) nor the whole syb2 had any effect on syntaxin clustering beneath granules, suggesting that synaptobrevin does not compete with the endogenous granular docking counterpart (Fig. 1C,D). In contrast, the cytoplasmic domain of syntaxin (1–243 amino acids, expressed via the same strategy using syxCD IRES Syx-GFP construct) had a dramatic effect on syntaxin cluster formation beneath granules: it was reduced several fold (Fig. 1C,D), similar to what has been observed previously^28^. Introduction of a mutation (I233A) that strongly reduces the interaction of syntaxin with munc18^29,30^ nearly abolished that effect (Fig. 1C,D), suggesting that interactions between syntaxin and munc18 are instrumental to the mechanism of docking.

### Syntaxin with a partial synaptobrevin 2 transmembrane domain strongly enhances syntaxin clustering on the plasma membrane

Next, I set out to test my hypothesis of whether syntaxin residing on granules and vesicles is the molecule that ties granules to the acceptor docking site consisting of the syntaxin cluster on the plasma membrane. To anchor more syntaxins to granules, I modified syntaxin transmembrane domain to match that of synaptobrevin 2. Synaptobrevin 2 is the best candidate, as it is one of the very few proteins that like syntaxin has a transmembrane domain (TMD) on its C-terminus, and it is predominantly found on granules and vesicles. Moreover, transmembrane domains of syntaxin and synaptobrevin 2 share some similarity: both have positively charged lysines and arginines in the juxtamembrane region and poly-isoleucine domains close to the C-terminal end. The last 5 amino acids (…VYFST) on the C-terminal end of synaptobrevin 2 appear important for targeting of this protein to granules. Particularly, syb2 mutant with most of its TMD replaced except for …VYFST rescued secretion in v-SNARE knock-out chromaffin cells to levels of wild-type synaptobrevin 2, which would be impossible without proper targeting of the mutant to granules^31^. Therefore, the ASTIGGIF sequence after the poly-isoleucine domain at the C-end of syntaxin was replaced with VYFST, generating a so-called vSyx construct (C-terminal end sequence: …VILGIII***VYFST***). To express vSyx such that its levels do not exceed that of syx-GFP, the coding sequence was placed in NPY-mCherry vector in the second reading frame (NPY-mCherry IRES vSyx, Supplementary Fig. 1)

**Figure 2:**
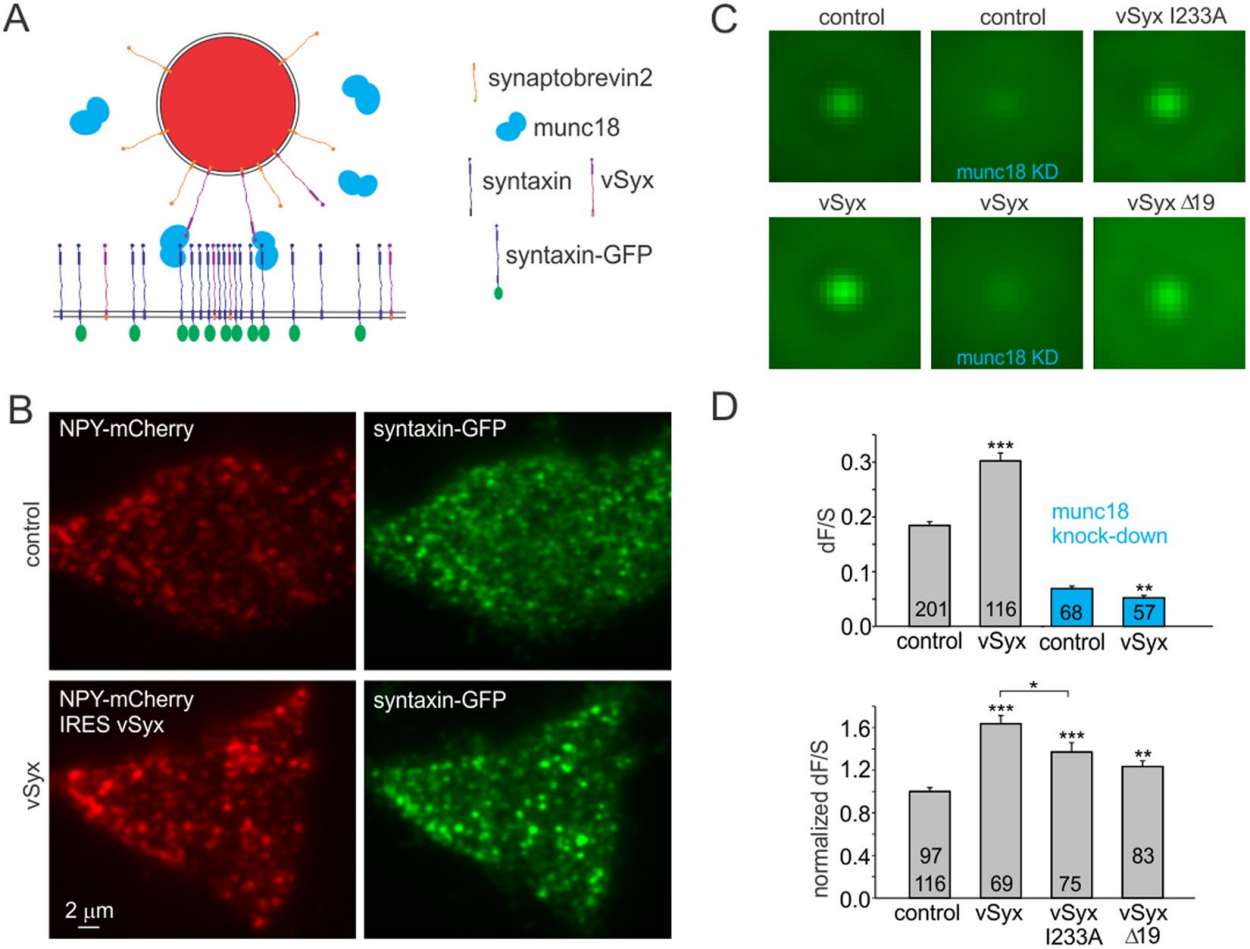
Syntaxin-GFP cluster formation is enhanced by vSyx mutant syntaxin. (**A**) Schematic molecular model as in Fig. 1A showing vSyx mutant residing on both granules (red) and the plasma membrane. (**B**) Exemplary dual-color TIRF images of cells expressing NPY-mCherry IRES vSyx and Syx-GFP (top panels, scaling: 1800–3500) and controls expressing NPY-mCherry and Syx-GFP (bottom panel). Cells were matched by Syx-GFP expression levels (control cell #1302-17, S-bkg = 1555; dF/S = 0.15; vSyx cell #1333-24, S-bkg = 1451; dF/S = 0.31). (**C**) Averaged Syx-GFP fluorescence beneath granules (scaled 2300–3700). Expression of vSyx mutant enhances Syx-GFP fluorescence (left panels); the effect is gone in the absence of munc18 (middle panels). Mutations of vSyx I233A and Δ19 diminished the phenotype (right panels). (**D**) Quantification of Syx-GFP cluster formation (dF/S) for conditions shown in **c**. vSyx enhancement of dF/S is gone in the absence of munc18 (blue bars). The number of cells included in the analysis is indicated inside the bars. 97 cells served as controls for vSyx Δ19 and 116 control cells for the rest of the mutants.

PC-12 cells expressing vSyx (NPY-mCherry IRES vSyx) showed much brighter syntaxin-GFP clusters than control cells expressing IRES Syx-GFP and NPY-mCherry, as depicted by the exemplary TIRF images (Fig. 2B) as well as by the averaged syx-GFP clusters beneath granules (Fig. 2C, left panels). The dF/S analysis revealed a highly significant enhancement of syx-GFP cluster formation of over 60% by vSyx (p < 0.00001, Fig. 2D). This data corroborates my hypothesis and also indicates that the syntaxin clusters can be enhanced, which was not previously achieved with any other manipulation. To date, only dispersion or reduction of syntaxin cluster on the plasma membrane was observed in response to membrane lipid content manipulation, syntaxin mutation or excess levels of unlabeled or GFP tagged syntaxin^11,12^ (Supplementary Fig. 1).

### Interactions with munc18 are critical for the enhancement of molecular docking

I next tested if munc18 is critical for the observed phenotype. I expressed NPY-mCherry IRES vSyx and IRES Syx-GFP in PC-12 cells with double knock-down of munc18-1 and 2^32^. The phenotype of syntaxin cluster enhancement by the vSyx was entirely gone in these cells (Fig. 2C,D). In the absence of munc18, syntaxin clusters beneath granules were greatly reduced, as evidenced by the averaged syx-GFP beneath granules as well as dF/S measurement, confirming that munc18 is critical for molecular docking of secretory granules. The dF/S value for vSyx was furthermore reduced by about 25%, likely due to vSyx diluting the Syx-GFP probe.

I performed analysis of different mutants of vSyx to elucidate the domains and interactions critical for enhancement of docking by vSyx, focusing on syntaxin – munc18 interactions. Surprisingly, the vSyx with an I233A mutation that abolishes interaction with munc18 *in vitro* ^30^ had only a moderate effect (Fig. 2C,D). In contrary, deletion of the N-peptide (19 amino acids at the N-terminal end, Δ19) from the vSyx mutant eliminated most of the enhancement of syntaxin clusters, suggesting that the N-peptide of syntaxin has an important role in attaching granules to syntaxin clusters on the plasma membrane (Fig. 2C,D). In combination with the crystal structure of syntaxin-munc18 complex, my latter observation suggests that the N-peptide of vSyx is binding to the syntaxin-munc18 complex on the plasma membrane. Indeed, the N-peptide binding site is segregated, and syntaxin and munc18 form a stable complex even without the N-peptide^19^. The expression of unlabeled proteins was validated using immunocytochistry and western blotting (Supplementary Figures 2 and 3).

### vSyx forms clusters beneath granules like syntaxin-GFP with a small fraction residing in granules

**Figure 3:**
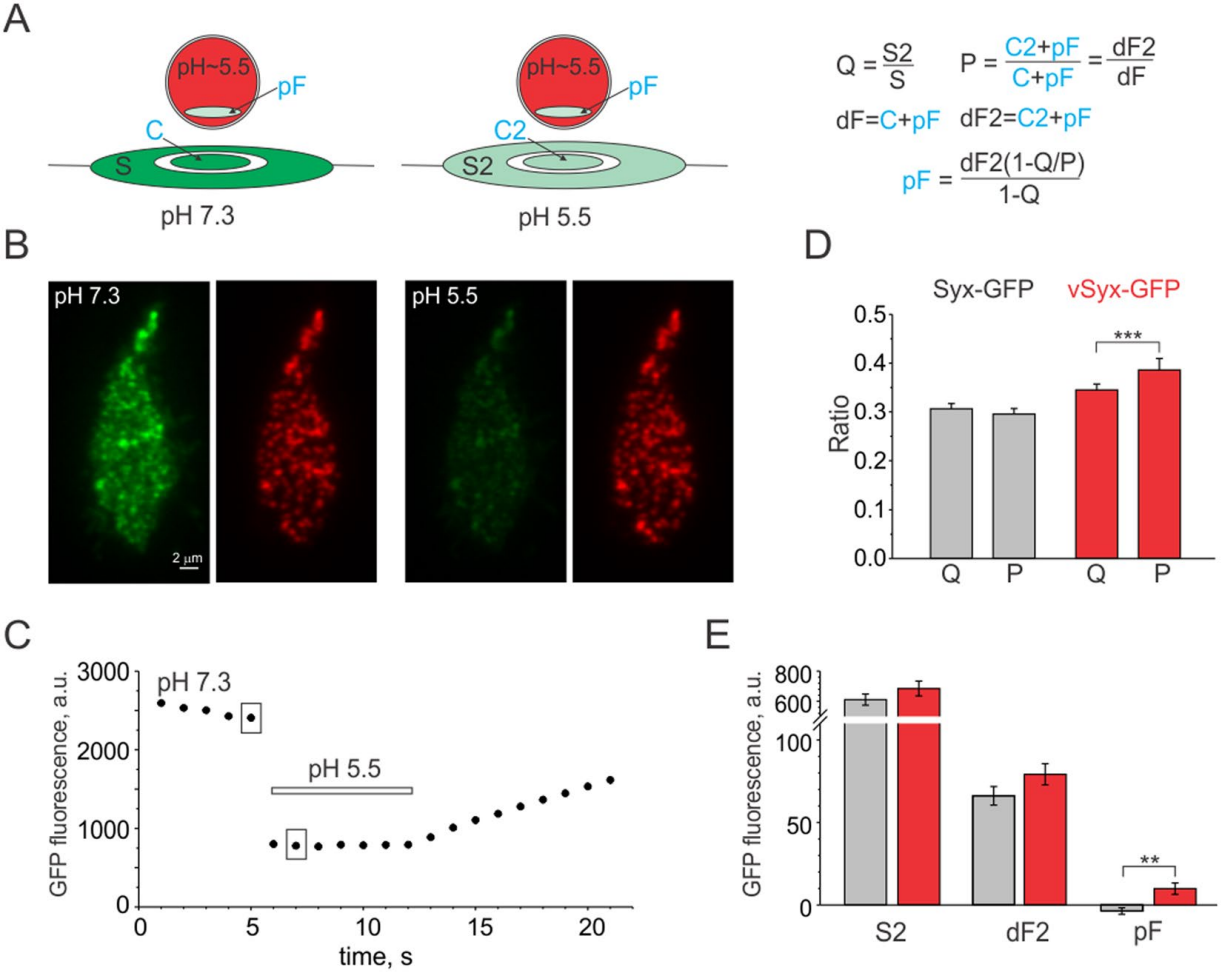
Quantification of vSyx-GFP fluorescence in granules. (**A**) Schematic representation of vSyx-GFP signal consisting of fluorescence in a granule-free zone (annulus, S), cluster beneath granule (C) and quenched GFP fluorescence (pF) inside the NPY-mCherry labeled acidic granule (red). Perfusion with low pH solution, which quenches GFP fluorescence on the plasma membrane, allows calculation of pH insensitive fluorescence (pF) inside granules (right panel). S, S2, dF and dF2 are measured (black), while C, C2 and pF can be calculated (blue). (**B**) Exemplary recording of a cell expressing vSyx-GFP (cell #3521) before and after perfusion with solution of pH5.5 (same scaling: 1800-900 GFP; 1800-1200 mCherry). (**C**) Whole footprint GFP fluorescence intensity of the cell #3521; 5 images were taken prior to the onset of the 7- second perfusion (horizontal bar). Boxed points represent images shown in B, which were used for analysis. (**D**) Quenching of the annulus signal (Q) and of the signal directly beneath granules (P) is almost the same for Syx-GFP (gray, n = 41; 1263 granules); while for vSyx-GFP (red, n = 46, 1354 granules), the P ratio is statistically significantly higher (p < 0.00001) indicative of a GFP fluorescence fraction that is insensitive to pH change. (**E**) Averaged values of the signal in the annulus and cluster are not significantly different between Syx-GFP and vSyx-GFP, while pH insensitive fluorescence is statistically significantly higher for vSyx-GFP (red).

To confirm that the vSyx phenotype is indeed due to targeting of this protein to granules, we generated a vSyx linked to GFP to characterize its localization. Filling secretory granules with neurotransmitters utilizes V-ATPase, resulting in a low pH of 5.5 inside granules^33,34^. GFP fluorescence intensity is sensitive to pH, being quenched significantly at low pH, like in secretory granules and vesicles^35^. I used this property of GFP to determine what fraction of vSyx-GFP is quenched inside granules. GFP is linked to syntaxin and vSyx on the C-terminus, such that GFP is exterior to the cell if syntaxin is residing on the plasma membrane, and inside a granule if it is anchored to a granule (Fig. 2A). My approach was based on the assumption that GFP fluorescence in a granule-free zone around the granule (annulus, S) is coming solely from syntaxin on the plasma membrane, while GFP fluorescence beneath a solitary granule (F) consists of syntaxin cluster (C) and quenched GFP signal from inside the granule (pF, Fig. 3A). Lowering external pH will quench exterior GFP and leave GFP inside granules unchanged (pF = protected fluorescence), which can be calculated by the formula in Fig. 3A, which is explained in detail in Supplementary Material.

Perfusion of cells with external solution at pH 5.5 led to considerable dimming of a GFP signal in a whole cell footprint for both Syx-GFP and vSyx-GFP in less than 2 seconds (Fig. 3B,C). Annulus and fluorescence beneath solitary granules, which were present in both images in normal pH = 7.3 (S, F) and low pH = 5.5 (S2, F2) were determined similar to the dF/S analysis. Fluorescence dimming in the annular area (Q = S2/S ratio, Fig. 3D) was almost identical to fluorescence dimming beneath granule (P = dF2/dF, Fig. 3D) for syx-GFP. In contrast, vSyx-GFP fluorescence beneath granules dimmed less than in the annulus (Fig. 3D), indicating the presence of pH insensitive fluorescence, likely located inside the granules. Calculation of protected GFP fluorescence (pF) revealed a small fraction of 13 ± 4.5% of dF2 value or 9.9 ± 3.4 absolute value of quenched GFP for vSyx-GFP, which was statistically significantly higher than for Syx-GFP (p < 0.0023, Fig. 3E). I failed to detect protected GFP fluorescence for Syx-GFP, likely because it is below the detection limit of this approach. It is important to mention that GFP fluorescence inside granules is likely reduced due to FRET interactions with mCherry. Moreover, although I tried to minimize diffusion of Syx-GFP and vSyx-GFP by choosing images only 2 seconds apart, as well as compensating for granule movement by restricting analysis to granules present in the same location before and after pH change, those factors still likely contributed to underestimation of protected GFP fluorescence (pF). For example, it is impossible to account for syx-GFP diffusion away from the center to the annulus due to cluster mobility. Moreover, a granule moving upward away from the coverslip surface will produce an exponential decrease in the already low quenched GFP fluorescence coming from inside the granule^12,36^. These factors likely underlie the resulting small negative value in pF obtained for Syx-GFP.

An alternative approach of dequenching granule-resident GFP using ammonium chloride solution was attempted, but was not optimal due to the longer time required to penetrate through the cytoplasm and granules (5–10 seconds) causing asynchronous brightening and bleaching of the low vSyx-GFP signal inside granules.

An exemplary cell in Fig. 3B shows clustered vSyx-GFP pattern, which looks similar to that of Syx-GFP (Figs 1B and 2B). Consistent with that, dF/S values for Syx-GFP and vSyx-GFP were very similar: 0.12 ± 0.01 (n = 38) and 0.14 ± 0.01 (n = 48), respectively (p = 0.13). Thus, the vSyx mutant behaves like wildtype syntaxin but with a small fraction of the vSyx residing in the granules.

Syntaxin cluster was estimated to consist of 75–90 molecules^13,26^. 13 ± 4.5% would give an estimate for vSyx in granules of about 10-11 molecules. Alternative estimation can be made from absolute quenched GFP values (pF) of 9.9 ± 3.4 for vSyx-GFP. External GFP was dimmed to about 30% (Q ratio) after perfusion with external solution with pH 5.5. Assuming that pH in granules is 5.5, pF for vSyx-GFP can be calculated to be 33 ± 11 at pH 7.3. GFP intensity of 3.4 represents a density of 1 fluorescent GFP molecule^13^ (Supplementary Material) giving an estimate of 9.7 ± 3.3 vSyx molecules per granule.

### vSyx facilitates morphological attachment of granules to the plasma membrane

**Figure 4:**
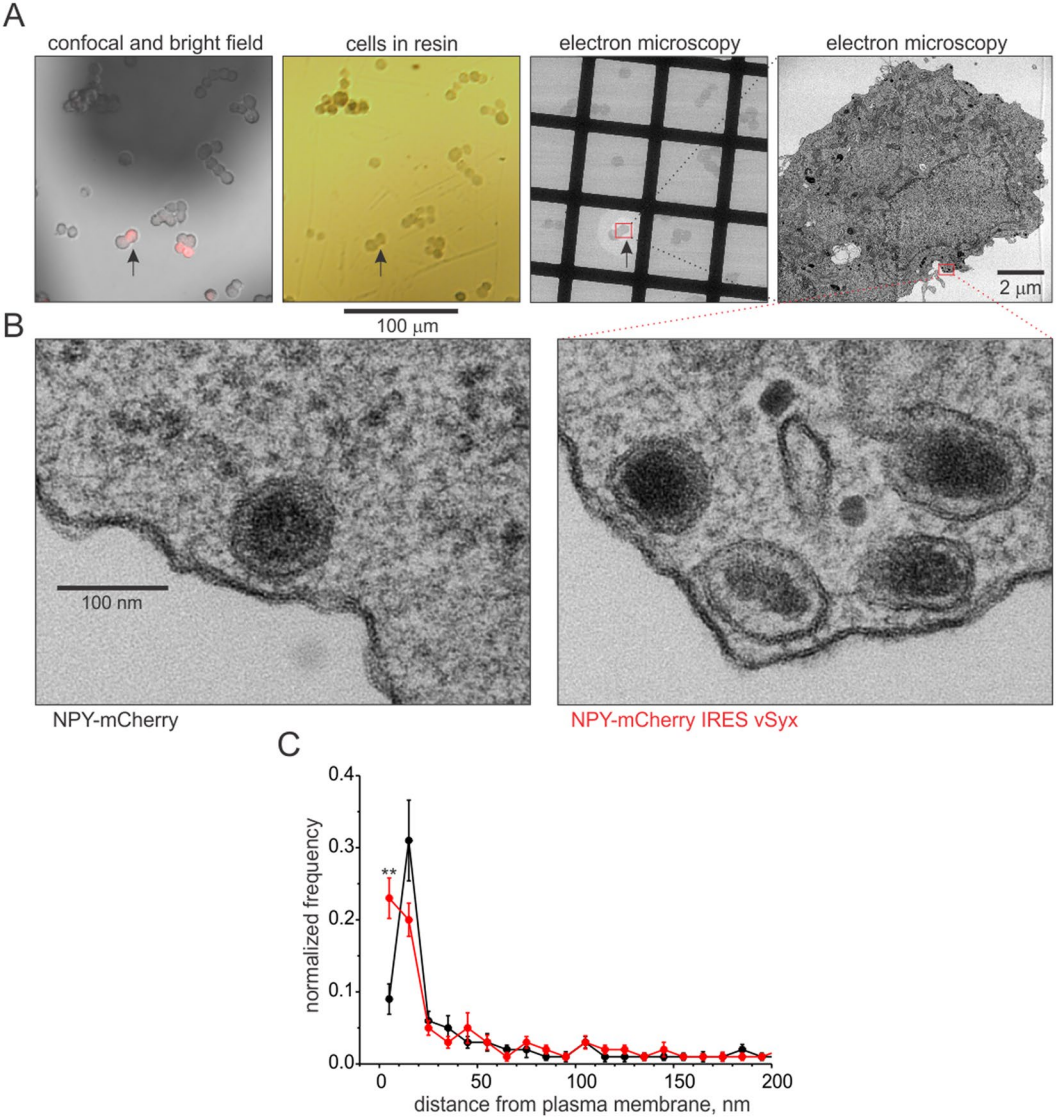
Electron microscopy of PC-12 cells expressing NPY-mCherry IRES vSyx. (**A**) Exemplary identification of the transfected cell (#5347, arrowhead). Fluorescent cells were identified with confocal and light microscopy and subsequently re-identified after embedding in resin. Low resolution electron microscopy image (115x) shows preserved location of the transfected cell (arrowhead) magnified at 2900x (right panel). See Materials and Methods for more details. (**B**) Exemplary image showing granules from a small boxed area in the cell in A (49,000x magnification, right panel), as well as exemplary image of a granule in control cell expressing NPY-mCherry (left panel). (**C**) Cells expressing NPY-mCherry IRES vSyx (red, n = 13, 477 granules) had significantly more granules in the immediate proximity to the plasma membrane within 10 nm (p = 0.001), compared to cells expressing NPY-mCherry (black, n = 14, 358 granules). Distribution represents the averaged normalized distribution with bins of 10 nm per each cell, with error bars representing SEM.

To test whether enhancement of molecular docking by vSyx mutant changes morphological attachment of granules to the plasma membrane, I implemented electron microscopy. For this, correlative light and transmission electron microscopy was performed: cells expressing NPY-mCherry (controls) and NPY-mCherry IRES vSyx were imaged with confocal imaging to identify transfected cells, which were later re-identified in electron microscopy based on their location as illustrated in Fig. 4A. Indeed, the data analysis of the distance between granules and the plasma membrane revealed more granules in the immediate proximity to the plasma membrane (<10 nm, p = 0.001) when vSyx was present (Fig. 4C). Interestingly, I observed a visually larger surface of contact between granule membrane and plasma membrane with bridge-like connections between granules and plasma membrane in the presence of vSyx, as depicted by the inset image (Fig. 4B).

In view of my hypothesis, the enhancement of docking in the presence of vSyx can be interpreted as follows: if a granule with more syntaxins on its surface can attach to a plasma membrane at more points, the probability to finding the granule closer to the plasma membrane will be higher (Fig. 4C). Granules with more syntaxins on their surface but farther away than the distances at which molecular docking interactions can occur likely have no advantage over control granules with fewer syntaxin molecules to mediate docking. Looking at electron microscopy of docking with resolution of less than 5 nm revealed phenotypes of synaptic proteins not previously detected due to limitations in methods and resolution, thus emphasizing the importance of looking at small distances to reveal molecular interactions between granules and plasma membrane^17^. Taken together, these data confirm the correlation between *molecular* docking measured by assaying syntaxin clusters beneath granules, and *morphological* docking assessed by ultrastructural analysis.

## Discussion

Following the discovery that botulinum toxins block neurotransmission by cleaving of SNARE proteins, SNAREs were acknowledged to be the central molecular components of vesicular fusion not only at synapses but in almost all eukaryotic cells. The regulatory elements that make neurotransmission highly tunable and very precise in time and space are still debated. It is widely accepted that SNAREs constitute the core fusion machinery with accessory proteins like munc18, munc13, synaptotagmin and complexin modulating SNARE proteins; full assembly of which completes membrane merger upon stimulus^37^. It was hypothesized that partial assembly of SNAREs corresponds to different preparatory steps vesicles undertake prior to fusion^38^.

By targeting few copies of syntaxin molecules to granules I discovered a new function of syntaxin in mediating docking of granules. Although unexpected, this finding does not contradict previous observations, but, rather, helps arranging those in a more comprehensive picture, opening up new possibilities for understanding and manipulating neurotransmission on a molecular level. I demonstrated that the N-peptide of syntaxin is critical for syntaxin’s role in mediating granule docking from a granule side. In support of this finding, it was shown that the functional role of the N-terminal end of syntaxin could be uncoupled from the rest of syntaxin^39^. In particular, when syntaxin was split in two parts, the N-terminal end and the rest of syntaxin containing SNARE domain, only expression of the two parts together rescued development and neurotransmission in nematodes with genetic deletion of syntaxin.

Early on it was demonstrated that syntaxin1A does not bind synaptobrevin in the absence of SNAP-25^40^. This observation, in the view of my findings, makes a lot of sense together with the data showing that there is almost no SNAP-25 found on vesicles^21^; vesicular syntaxin is very unlikely to engage in SNARE complex formation before the vesicle reaches SNAP-25 on the plasma membrane.

One valuable aspect of my work is the demonstration that a small increase in the number of syntaxin on granules leads to profound effects in granule docking. Syntaxin cluster was estimated to consist of 75–90 molecules^13,26^, but only about 6 syntaxins were found on synaptic vesicles^21^. A coarse estimate on the number of vSyx in granules is about 10 copies. When compared to overall syntaxin levels on the plasma membrane, the number of vSyx in granules was close to 2%. Increase of syntaxins on granules by 10 or fewer copies lead to a profound enhancement of docking, suggesting quantal molecular mechanism. Although syntaxin was shown to be residing on neurosecretory granules, its levels were not quantified. It is possible that the number of vSyx copies does not exceed the endogenous syntaxin levels in granules, leading to 60% increase in syntaxin-GFP cluster brightness beneath granules.

Interestingly, the other major isoform of syntaxin, syntaxin1B was predominantly found on granules in chromaffin cells^20,41^. Syntaxin1A and Syntaxin1B are functionally interchangeable, as syntaxin1A KO is viable and only removal of both isoforms leads to embryonic lethality^42,43^. Moreover Syntaxin1B but not Syntaxin1A was implied to be necessary in synaptic transmission^43^. Although little is known on how these isoforms support docking and fusion in chromaffin cells, the fact that syntaxin1B can substitute for syntaxin1A suggests that granule-bound isoform is sufficient to support docking and fusion of secretory granules.

It is tempting to hypothesize that the number of SNAREs per vesicle or availability of those proteins would control fusion probability. However, a huge discrepancy of over 10-fold was observed in the number of SNARE proteins found on vesicles and the number of SNARE complexes per fusion event. SNAP-25, syntaxin and synaptobrevin are highly abundant with over 70 molecules per vesicle and per fusion site^13,21,44^. By comparison, the number of SNARE complexes was estimated to be surprisingly low: 1–3^45,46^. The titration experiments showed that lowering syntaxin expression levels led to gradual decrease in release efficiency in hippocampal neurons, suggesting that the number of syntaxins correlates with fusion efficiency^47^. In contrary, changing the expression levels of synaptobrevin or SNAP-25 does not seem to have any effect on exocytosis^16,48^. Despite the fact that a highly conserved family of proteins is mediating exocytosis, the presynaptic release probability and overall properties of neurotransmitter release are highly variable and at least in part underlie presynaptic plasticity. I speculate that the number of syntaxin molecules on vesicles and granules is a molecular rate-limiting step, controlling the number of SNARE complexes per fusion and conceivably may determine release probability. Such a mechanism of SNARE mediated vesicle fusion would open up a variety of possibilities of how neurotransmission is quantitatively regulated on a molecular level. Discovering the mechanism that controls the number of syntaxins on vesicles and their availability for SNARE complex interactions will likely be a key to manipulation of neurotransmitter release.

Based on the data I propose a model in which initial docking of granules is mediated by munc18 bridging N-terminal end of granules’ syntaxin to syntaxin on the plasma membrane. Subsequent molecular steps possibly involve engagement of SNAP-25 – syntaxin complex in 1:2 stoichiometry; this complex was found to be a stable intermediate prior to ternary SNARE complex assembly with synaptobrevin at 1:1:1 stoichiometry^49^. Fusion is likely completed rapidly, in an all-or-none fashion, because of the abundance of synaptobrevin molecules, which readily assemble into SNARE complexes upon stimulus. The proposed molecular mechanism makes sense from both the molecular aspect as well as energetic efficiency. Instead of partial assembly of SNAREs underlying subsequent preparatory steps in vesicle fusion I propose that different complexes involving SNAREs and accessory proteins are responsible for docking and fusion. However, the exact sequence of molecular events, taking place after munc18 bridging syntaxins on opposing membranes leading to membrane fusion, still remains to be investigated in detail.

Another interesting aspect of syntaxin-munc18 interaction is that syntaxin expression levels are reduced in the absence of munc18 and vice versa^6,25^. I speculate that syntaxin-munc18 mediated docking leading to fusion is one of the pathways for syntaxin to get to the plasma membrane from granules. With more syntaxins on the plasma membrane the probability of new docking and fusion events is likely increased, which may serve as a positive feedback mechanism for exocytosis. Moreover, such mechanism may be a molecular ‘counter’ of prior fusion events.

Taken together, the newly-discovered mechanism of syntaxin-munc18 mediated docking advances our understanding of the molecular mechanism of neurotransmission, and paves the way for molecular quantification and precise manipulation of neurotransmitter release. This finding has broad implications as syntaxins on granules likely control the initial step in vesicle availability for fusion and conceivably mediate the probability of release. Enhancement of this pathway can be instrumental to treating neurological disorders where an insufficient amount of neurotransmitter is released, like Parkinson’s disease.

## Materials and Methods

### Cell culture and electroporation

PC-12 cell lines were generously provided by Thomas Martin (University of Wisconsin-Madison). Cells were maintained in T25 flasks (Nalgene) at 37 °C, 10% CO_2_ in DMEM (High Glucose, Gibco) supplemented with 5% bovine calf and 5% horse serum (Hyclone). PC-12 cell line with 2 munc18 isoforms (DKD7) knockdown was generously provided by Shuzo Sugita (University of Toronto). DKD7 cells’ media was supplemented with 6% of serums, 2.5 μg/ml of puromycin, 400 μg/ml of G418 and 2.5 μg/ml plasmocin (InvivoGen). After transfection with the Neon electroporation system (Invitrogen), cells were plated on Poly-L-Lysine (Gibco) coated coverslips. The amounts of DNA per 100 μl were 1-2 μg for NPY-mCherry, Syx-GFP and vSyxGFP plasmids, and 15 μg for constructs containing IRES syxGFP. Imaging was performed 20–26 hours after transfection at room temperature. Cells were used on passages 17 through 30.

### Plasmids

NPY-mCherry and Syx-GFP constructs were described by Barg *et al*., 2010. IRES Syntaxin-GFP construct was kindly provided by Xi Chen. The GGSGGSGGSGGS flexible linker was inserted between Syntaxin (or vSyx) and GFP, in Syx-GFP and vSyx-GFP constructs. Synaptobrevin and syntaxin mutants’ expression at levels higher than syntaxin-GFP was achieved by placing the coding sequences of unlabeled mutants in the first reading frame of the bicistronic vector, followed by IRES Syx-GFP^50^. Syx1-243 IRES Syx-GFP (syxCD) and Syx 1–243 I233A IRES Syx-GFP (SyxCDI233A) were generated by Biobasic (Canada). Syb2 IRES Syx-GFP (Syb2), Syb2 1–96 IRES Syx-GFP (Syb2CD), vSyx plasmids (NPY-mCherry IRES vSyx) and its mutants were made by Pronovus Bioscience (Mountain View, CA) through Science Exchange. All constructs contained the CMV promoter and Kozak sequence before each reading frame.

### Fluorescence microscopy

Simultaneous dual-color TIRF microscopy was conducted as previously described by Barg *et al*., 2010. For dF/S experiments, 488 nm and 568 nm laser intensities were set to 10 mW and 2 mW, respectively. Cells were found by visualizing NPY-mCherry labeled granules with 568 nm excitation. After adjusting focal plane, simultaneous GFP and mCherry signals were acquired (256 × 512 pxl). For dF/S analysis 10 images (20 ms exposure) were acquired at 16-bit resolution and averaged per cell. The external solution contained (in mM) 140 NaCl, 2.8 KCl, 2.5 CaCl_2_, 1 MgCl_2_, 5 HEPES, and 30 Glucose at pH 7.3. Cells expressing control plasmid were imaged in parallel for each experimental condition; data was acquired on at least 2 different cultures of cells for each experimental condition.

### Analysis of TIRF data

Images were analyzed with MetaMorph (Molecular probes) and MatLab (MathWorks) using custom routines. Alignment of red and green channels and dF/S analysis was conducted as previously described^12^ with the following modifications. Granules were detected automatically in the MetaMorph software after using a top-hat filter (detection threshold was set to 30); only solitary granules that did not have any neighboring granules in a radius of 6 pixels from the center were included. The 9 × 9 pixel images of granules were excised and analyzed with Matlab by fitting each granule with a 2D Gaussian formula superimposed on a single plane slope:

$$f(x,y)=Cx+Dy+offset+Aexp(-\frac{{({\rm{x}}-{\rm{x}}0)}^{2}+{({\rm{y}}-{\rm{y}}0)}^{2}}{2{\sigma }^{2}})$$

To ensure that the red fluorescent puncta represent single immobile granules, only granules with a 2D Gaussian fit that met the following criteria were included: *offset* < 4000; distance from center $(\sqrt{x{0}^{2}+y{0}^{2}}) < 1.25{\rm{pxl}}$; full width at half maximum (FWHM = σ2.35482) 1.6 < FWHM < 4.25pxl; amplitude A > (*offset*-1800)/2. The 1800 value is a typical value for background fluorescence. Only cells with 8 or more qualifying granules were included in analysis. The dF/S was calculated by the formula shown in Fig. 1B; background fluorescence was determined for each image. Cells with S values ranging from 400–2000 and dF standard error below 10% of the S-value were included in analysis. Averaged images of Syx-GFP clusters were generated by averaging all the vesicles and clusters beneath vesicles in cells that passed all the criteria.

### Low pH solution perfusion

The low pH external solution contained (in mM) 120 NaCl, 2.8 KCl, 2.5 CaCl_2_, 1 MgCl_2_, 30 MES Na-salt, 5 glucose at pH 5.5. A thin-walled glass capillary was prepared on a puller (Sutter) to generate a pipette with an opening of a few microns. To initiate perfusion, slight positive pressure was applied using a Picospritzer (Parker). The 488 and 568 nm lasers were both set to 2 mW intensity to reduce bleaching of GFP. A time lapse at 1 Hz with 100 ms exposure was taken with 5 image acquisitions, prior to low pH solution perfusion. For every cell, whole cell fluorescence was plotted against time to choose 2 images 2 seconds apart for analysis, which was performed similarly as for dF/S measurements. Only solitary granules that did not have any other granules in a radius of 4.5 pxl from the center were included. To restrict analysis to immobile granules, granules that passed Matlab 2D Gaussian fit exclusion criteria in the first image (pH 7.3) were fitted again in the second image (pH 5.5). Only granules that passed the above mentioned exclusion criteria in both images were included in analysis. Cells with Q ratio <0.67 and S value ranging from 1000 to 5000 of GFP fluorescence intensity were included in analysis. Photobleaching of quenched GFP was quantified to be 1% per one image acquisition; pF values were corrected for quenched GFP bleaching. The dF/S measurements on this data were obtained from cells with S value ranging from 400–2000.

### Electron microscopy

Colocalization light and electron microscopy was conducted similar to as previously described^51^. PC-12 cells expressing NPY-mCherry and NPY-mCherry IRES vSyx were compared. Cells were transfected and plated on poly-l-lysine coated Aclar coverslips with a printed grid to facilitate site recognition. Approximately 24 hours after transfection, cells were fixed with ice-cold 1.5% glutaraldehyde, 1.5% paraformaldehyde in 0.1 M sodium cacodylate buffer with 0.05 M sucrose and 0.25% CaCl_2_ pH 7.4. Cells were then imaged on a FluoView FV1000 (Olympus) confocal microscope to collect a bright field image, as well as mCherry fluorescence with a 20x objective. Cells were then microwave processed for electron microscopy using Biowave (Ted Pella): osmicated in 2% OsO_4_ reduced by 1.5% K_3_[Fe(CN)_6_], stained with 5% uranium acetate *en bloc*, serially dehydrated with ethanol and then embedded in EMbed-812 (Electron Microscopy Sciences). Embedded cells were reimaged to verify the location of the fluorescent cells. Ultrathin sections (60 nm, gray/silver interference color) were counterstained with 5% uranium acetate (8 min) and lead citrate (5 min) and imaged on a FEI Tecnai 12 BioTwin transmission electron microscope equipped with an AMT Active Vu-M 16 megapixel camera (Advanced Microscopy Techniques). Low magnification images of 60–115X were taken to confirm location of cells visualized with confocal imaging. Scaling to the same magnification and alignment of the confocal, bright field and low magnification electron microscopy was done for every cell. Analysis was done with the MetaMorph software (Molecular Devices). Granules were identified by the presence of a dense inner core. The closest distance from a granule to the plasma membrane was measured from micrographs (direct magnification 13,000x, final magnification ~1 nm/pxl). Cells with more than 12 granules located within 500 nm from the plasma membrane were included in analysis.

### Statistics

Student’s t-test was used as appropriate to assess statistical significance. For datasets that did not pass the Kolmogorov-Smirnov test for normality, the Mann-Whitney U test was used. Wilcoxon Signed-Rank test was used for comparing P and Q values in pH perfusion experiments. Significance was displayed at *p < 0.05, **p < 0.01, ***p < 0.001. Data are expressed as mean ± SEM.

## Electronic supplementary material

Supplementary information
