# Supplementary material for: Syntaxins on granules promote docking of granules via interactions with munc18
Source: Sci Rep. 2018 Jan 9;8:193. doi: 10.1038/s41598-017-18597-z (PMC5760731; doi:10.1038/s41598-017-18597-z)
Supplement: Supplementary file 1 — Supplementary information [file 41598_2017_18597_MOESM1_ESM.pdf]

Supplementary material

to the manuscript entitled

Syntaxins on granules promote docking of granules via interactions with munc18

by Maria Borisovska

Protein expression levels

The TIRF experimental setup allowed visualizing two colors, both of which were used to quantify syntaxin cluster formation beneath granules. Low expression levels of syntaxin-GFP were ensured by placing its coding sequence in the second reading frame of the bicistronic vector (IRES Syx-GFP, Supplementary Fig. 1a). The expression levels of syntaxin-GFP were tightly controlled by the fluorescence intensity; only cells with GFP fluorescence intensity ranging from 400 – 2000 in the footprint were taken as described in Materials and Methods. To minimize Syntaxin-GFP bleaching as it was expressed at low levels, cells were identified by NPY-mCherry fluorescence only. Image acquisition was performed after focal plane adjustment in the red channel. My experiments were conducted on the same TIRF imaging setup with the same settings as by Knowles et al., 2010 and Barg et al., 2010. The manuscript by Knowles et al., 2010 has a series of experiments measuring and calculating how many fluorescent units correspond to a fluorescent GFP molecule as well as endogenous syntaxin levels. In particular, GFP intensity of 1.7 represents a density of one GFP molecule per square micron at 0.1 mJ excitation (Table 1 in Knowles et al., 2010). Excitation used for the imaging experiments described here was two-fold higher: 0.2mJ (10mW x 20ms), thus intensity of 2 x 1.7 = 3.4 represents a density of one GFP molecule per square micron at 0.2mJ excitation. 400-2000 range is thus equivalent to 118 - 588 GFP molecules. Endogenous syntaxin was measured to be 540 molecules per square micron, giving the range of 0.22 to 1.09 fold more than endogenous syntaxin. On average, the syntaxin-GFP brightness per cell was about 900, corresponding to ~265 molecules, or 49% of the endogenous syntaxin level (Supplementary Fig. 1c).

Unlabeled proteins were expressed using bicistronic vectors paired with either syntaxin-GFP or NPY-mCherry, such that the expressing cells were identified either by presence of NPY-mCherry or syntaxin-GFP (Supplementary Fig. 1a). Protein expressed in the first reading frame is expressed at higher levels than the one after the IRES site. In order to determine the difference in expression levels between the proteins encoded in the first and a second reading frame, I expressed unlabeled syntaxin in the first reading frame and syntaxin-GFP in the second reading frame (Syntaxin IRES Syntaxin-GFP). dF/S measure of syntaxin-GFP clustering was reduced 2.845 fold when syntaxin was overexpressed, suggesting that the protein in the first reading frame is expressed at least 2.845 fold higher, resulting in dilution of syntaxin-GFP cluster beneath granules (Supplementary Fig. 1b). This result also serves as a control showing that increasing the numbers of unlabeled WT syntaxin does not lead to enhanced dF/S measure of molecular docking.

Lower amounts of DNA were used to express NPY-mCherry containing plasmids than IRES Syx-GFP containing plasmids (2 μg versus 15 μg) suggesting that unlabeled Protein Y expressed in NPY-mCherry plasmid was expressed at lower levels than IRES Syx-GFP. To confirm this, a single plasmid was expressed containing both: NPY-mCherry IRES Syx-GFP. Indeed, GFP intensity was the same on average, NPY-mCherry signal was brighter (Supplementary Fig. 1c).

Supplementary Figure 1d represents a summary of the protein expression ranges used for dF/S measurement. The 2.845 fold difference obtained in Supplementary Fig. 1b, a range for protein X expression levels can be calculated: 2.845x0.22 to 2.845x1.09.

Protein X: syb2, syb2CD, syxCD, syxCD I233A (Figure 1)

Protein Y: vSyx, vSyx I233A, vSyx Δ19 (Figure 2)

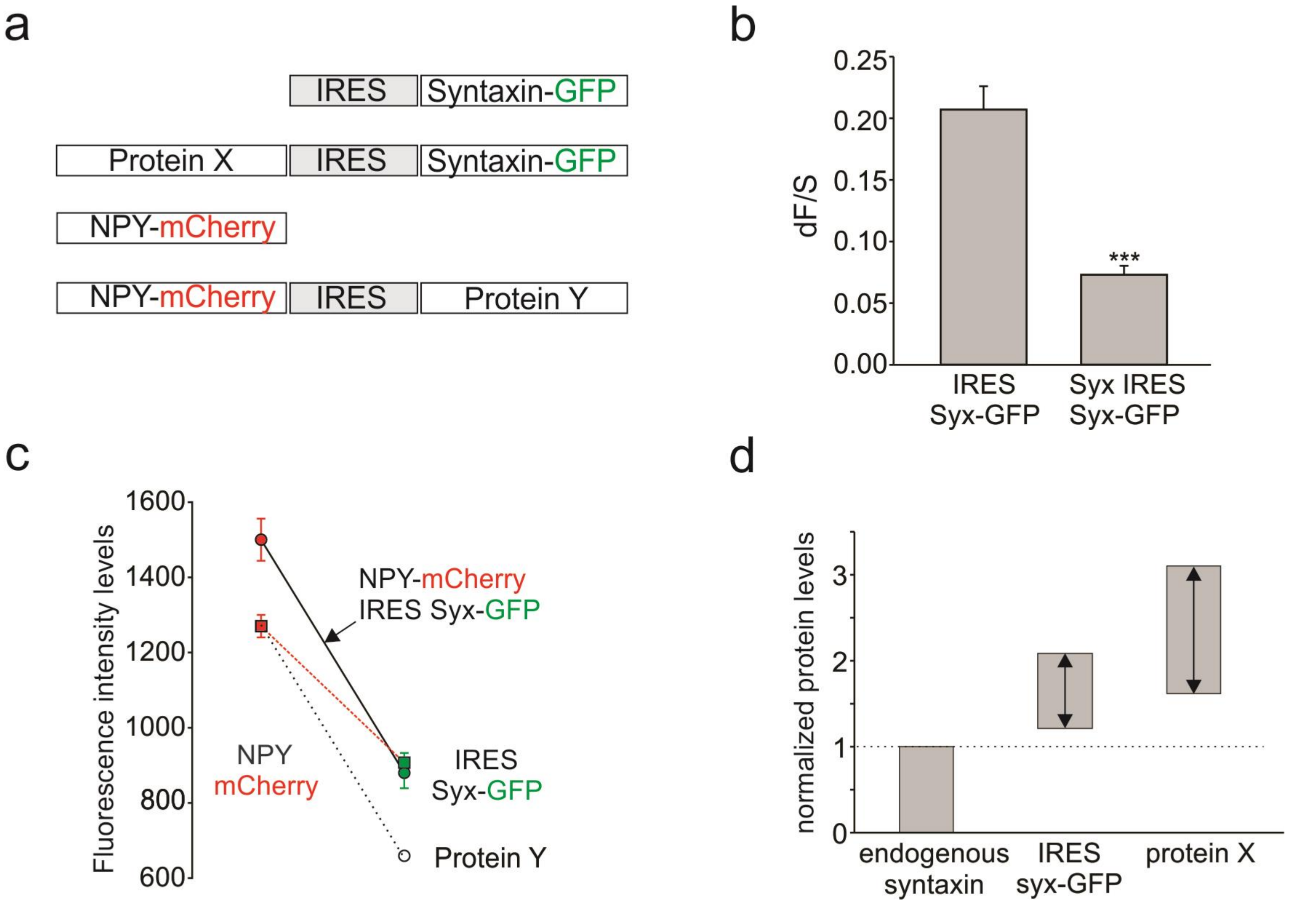

Supplementary Figure 1. Protein expression levels. (**a**) Schematic representation of the two plasmids used in this manuscript to express NPY-mCherry, syntaxin-GFP as well as unlabeled proteins. Two plasmids were used for protein expression. (**b**) Unlabeled syntaxin overexpression in the first reading frame (Syx IRES Syx-GFP, n=25) leads to a dramatic reduction of dF/S measure of syntaxin cluster formation beneath granules (p<3e-8) when compared to control (IRES Syx-GFP, n=23). (**c**) Correlation between NPY-mCherry fluorescence intensity and Syx-GFP fluorescence intensity. When NPY-mCherry and Syntaxin-GFP are expressed in the same plasmid (circles, arrowhead, n=69) or when 2 plasmids are co-expressed in the same cell (squares, dotted line, n=201 cells). Projected expression level of unlabeled Protein Y based on difference between NPY mCherry and Syntaxin-GFP expressed using the same plasmid (black dotted line). Note the normalization of the mCherry signal to GFP signal by multiplying the values by 5 to match the laser intensities set to 2mW for 568 nm and 10 mW for 488 nm. (**d**) Ranges of expressed proteins normalized to endogenous syntaxin levels. IRES Syx-GFP intensity range 400-2000 corresponds to 0.22 to 1.09 fold above the endogenous levels (dotted line). Protein X is expressed ~2.845 fold higher than IRES syx-GFP as evident from (b) resulting in range 0.62 to 3.1 fold higher than endogenous syntaxin.

Calculation of granule resident GFP

TIRF allows visualization of a two-dimensional image representing a granule (red) and a syntaxin cluster (green) viewed from the bottom of the coverslip, shown by horizontal line on the schematic drawing below (same as in Fig.3A). I measure S, a value of green fluorescence in the annulus surrounding a granule before and after low pH solution perfusion. I also measure dF, fluorescence just beneath a granule, which consists of C (cluster, inner circle) and pF (protected fluorescence inside granule above the cluster). dF= C+pF (Equation 1). In low pH solution the external GFP in the cluster is quenched, while protected fluorescence colocalizing with granules does not change. Therefore the green fluorescence just beneath a granule consists of a dimmed cluster plus unchanged protected fluorescence: dF2=C2+pF (Equation 2). The quenching factor Q is determined from how much the annulus area has dimmed in low pH solution compared to normal pH. Q=S2/S=C2/C (Equation 3). As cluster is also located on the plasma membrane it should quench exactly the same as annulus area. Quenching factor P is calculated for GFP Fluorescence beneath granules dF2/dF=P (Equation 4). The assumption is that there is no protected fluorescence in the area where there are no granules. If annulus area dims in response to low pH buffer the same as the area just beneath granule or if Q=P, it means that there is no protected fluorescence, as GFP in both areas quenches the same. If P is greater than Q, there is a fraction of dF fluorescence that is insensitive to external pH change, so called protected fluorescence (pF) or pF>0.

Taken together there are 4 equations with 3 unknown variables: C, C2 and pF. Q, P, dF, dF2 are obtained from the fluorescence measurements.

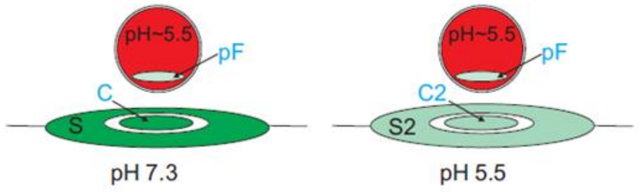

In the Equation 2:

dF2=C2+pF

Replace C2 with QC from Equation 3

dF2= QC +pF

Replace C with dF-pF from Equation 1

dF2= Q(dF-pF) +pF

dF2= QdF-QpF +pF

pF - QpF = dF2-QdF

pF(1-Q)= dF2-QdF

From the Equation 4 dF=dF2/P

pF(1-Q)= dF2-dF2(Q/P)

pF(1-Q)= dF2(1-Q/P)

pF= dF2(1-Q/P)/(1-Q) (final formula shown in Fig. 3A)

Verification of expression of unlabeled proteins

To confirm the expression of unlabeled proteins I employed immunocytochemistry and western blotting. The constructs were expressed in HEK293 cells because those cells are devoid of endogenous syntaxin and synaptobrevin, thus, enabling unequivocal visualization of the expressed unlabeled synaptobrevin2, syntaxin 1A and its mutants. For synaptobrevin2 and synaptobrevin2 cytoplasmic domain (syb2CD) mutant, only cells expressing syntaxin-GFP showed immunoreactivity for synaptobrevin antibody as depicted by exemplary micrographs (Supplementary Fig. 2a). Similarly, immunoreactivity for syntaxin antibody was observed for vSyx and its mutants only in cells also showing NPY-mCherry fluorescence (Supplementary Fig. 2b). For constructs encoding syntaxin cytoplasmic domain (syxCD) and I233A mutant (syxCD I233A) in the first reading frame and syntaxin GFP in the second reading frame, syntaxin antibody recognized both tagged and untagged syntaxin proteins. Therefore, to further confirm the expression of both syntaxin mutants, western blot analysis of HEK293 cells lysates using the same syntaxin antibody was employed (Supplementary Fig. 3b). A single band was observed between 25 and 35 kDa markers for both syntaxin mutants in good agreement with the calculated molecular weight for syntaxin 1-243 amino acids of 28.2 kDa. Retina served as a positive control and untransfected HEK293 cells as well as HEK293 cells transfected with syb2CD IRES Syx-GFP served as negative controls for this experiment. Note that the syxCD and syxCD I233A bands are dimmer than a band for control retina homogenate due to transfection efficiency of HEK293 cells. Moreover, Syntaxin-GFP expression levels were estimated to be several-fold lower than that of syxCD or syxCD I233A, becoming below the detection limit of this method.

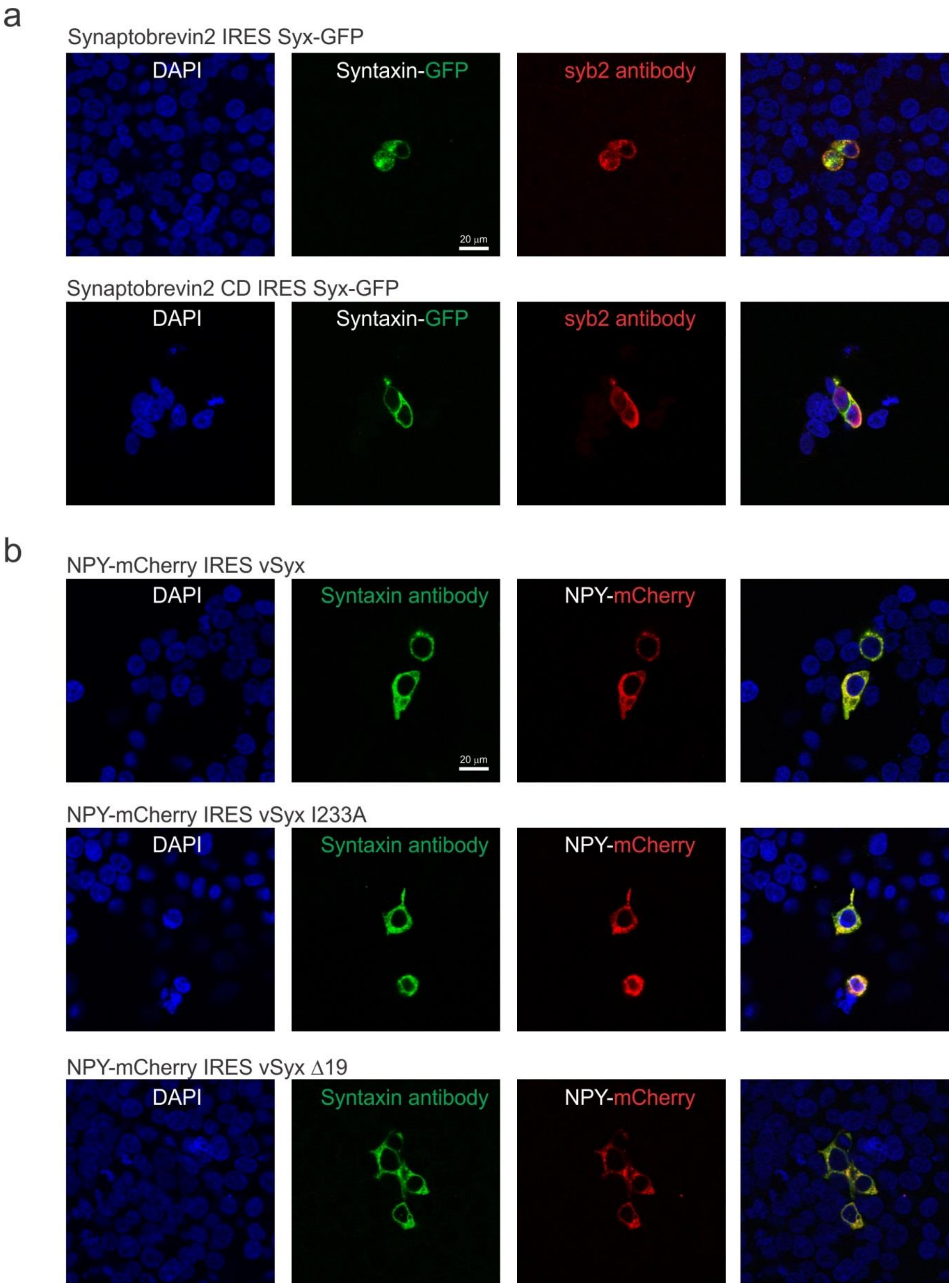

Supplementary Figure 2. Immunocytochemical visualization of unlabeled proteins encoded in constructs containing either syntaxin GFP or NPY-mCherry. (**a**) Representative confocal microscopy images of synaptobrevin2 immunostaining (red) of HEK293 cells expressing

synaptobrevin2 IRES syx-GFP (top) and HEK293 cells expressing cytoplasmic domain of synaptobrevin 2: syb2 CD IRES syx-GFP (bottom). (**b**) Representative confocal microscopy images of syntaxin immunostaining (green) performed on HEK293 cells expression vSyx (top), vSyx I233A (middle) and vSyx Δ19 (bottom) in constructs containing NPY mCherry (red) in the first reading frame.

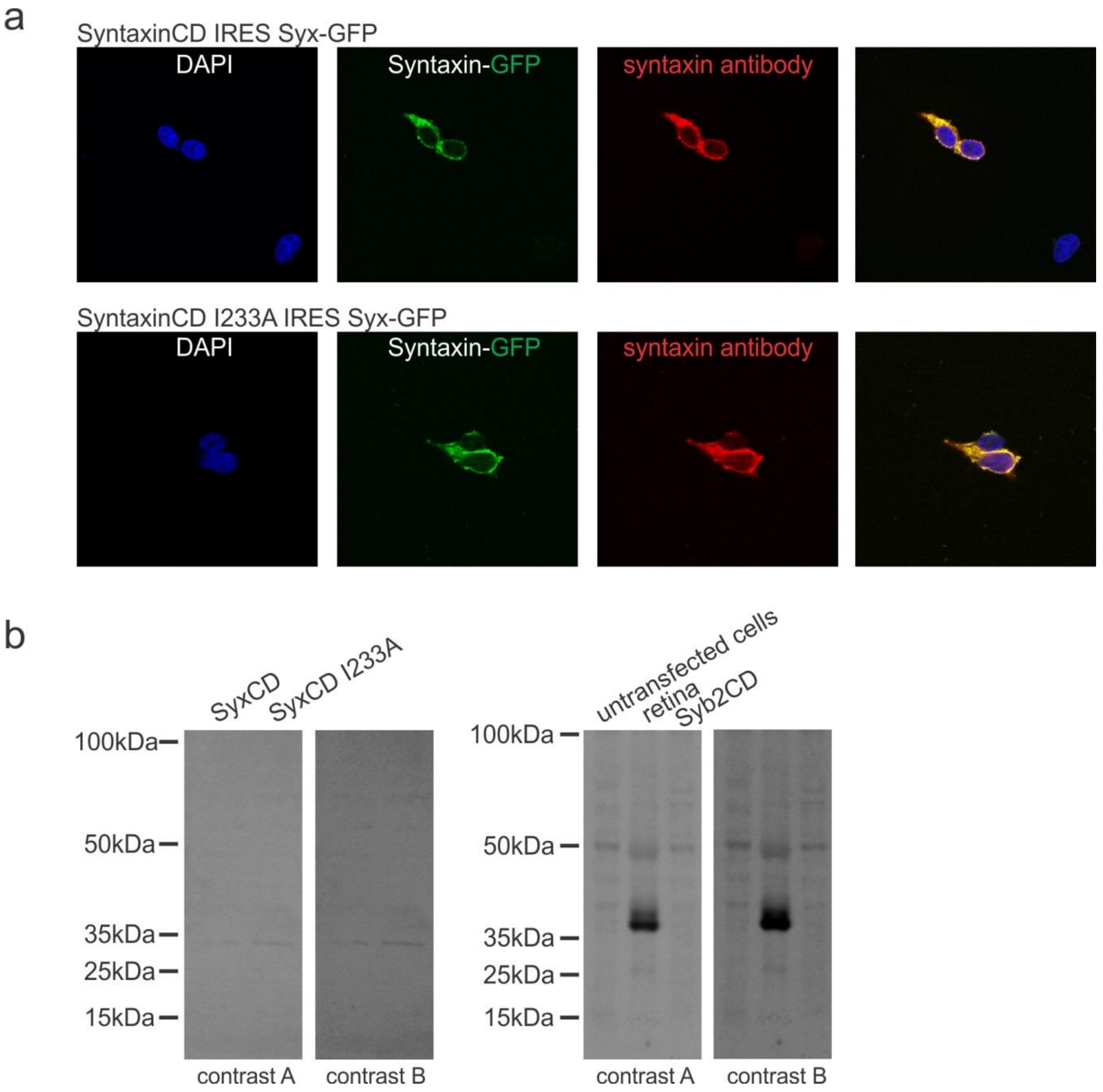

Supplementary Figure 3. Validation of expression of syntaxin cytoplasmic domain mutants (**a**) Immunocytochemistry of HEK293 cells expressing syntaxin CD IRES syntaxin-GFP (top) and syntaxin CD I233A mutant (syxCD I233A IRES syntaxin-GFP, bottom). (**b**) Western blot of HEK293 cells expressing both mutants showed immunoprecipitation and bands of the expected size for cytoplasmic domain mutants (~28 kDa, left panel). Retina homogenate, untransfected HEK293, as well as HEK293 cells transfected with syb2CD IRES Syx-GFP served as positive and negative controls, respectively. Both gels were photographed with the same settings. Contrast was linearly adjusted to 2-700 (Contrast A) and 2-208 (Contrast B) and displayed for both gels.

Supplementary Materials and Methods

Cell culture and transfection

HEK293 cells were cultured at 37°C 5%CO2 in DMEM (Gibco) media supplemented with 10% Calf Fetal Bovine Serum (Hyclone) and 1% Penicillin-Streptomycin. Transfections were performed using Effectene transfection reagent (Qiagen) using the manufacturer's recommended protocol. Cells were used for immunocytochemistry and western blotting 24-48 hours after transfection.

Immunocytochemistry

Immunocytochemistry and confocal imaging was performed as previously described (Borisovska et al., 2011; Morgans et al., 2006). Briefly, transfected cells were grown on poly-lysine coated coverslips, fixed in 4% paraformaldehyde, and labeled with antibodies against synaptobrevin 2 (rabbit polyclonal, 1:500; Synaptic Systems, Germany) or syntaxin 1 (clone HPC-1, 1:100), followed by anti-rabbit or anti-mouse secondary antibodies coupled to Alexa-594 (Molecular Probes, Eugene OR) or CY3 (Jackson ImmunoResearch Laboratories Inc., West Grove, PA). Images were acquired on a Leica TCS SP8 X confocal microscope using a 40x oil-emersion objective. Linear scaling was applied for all images.

Western blot

Lysates of transfected and untransfected HEK293 cells and mouse retina were prepared in RIPA buffer with the addition of a protease inhibitor cocktail (Cell Signaling Technology, Danvers, MA, USA). Lysates (~10ug protein per lane) were electrophoresed on precast 4%-12% polyacrylamide gradient gels (Novex; Invitrogen). The separated proteins were electrophoretically transferred to polyvinylidene difluoride (PVDF) membranes, which were then probed with a mouse monoclonal antibody to syntaxin 1 (clone HPC-1, 1:250) followed by anti-mouse IRDye 800CW secondary antibody (1:10,000; Li-Cor, Lincoln, NE, USA). Immunoreactive bands were visualized with an Odyssey infrared imaging system (Li-Cor). Images were processed using Image Studio Lite software.

References:

Barg, S., Knowles, M. K., Chen, X., Midorikawa, M. & Almers, W. Syntaxin clusters assemble reversibly at sites of secretory granules in live cells. *Proc. Natl. Acad. Sci.* **107,** 20804–20809 (2010).

Knowles, M. K. *et al.* Single secretory granules of live cells recruit syntaxin-1 and synaptosomal associated protein 25 (SNAP-25) in large copy numbers. *Proc. Natl. Acad. Sci.* **107,** 20810–20815 (2010).

Morgans, C.W., Ren, G. & Akileswaran, L. Localization of nyctalopin in the mammalian retina. Eur J Neurosci. **23,**1163-71(2006).
